# The Implementation of the Music and Memory Intervention in Nursing Facilities in Virginia

**DOI:** 10.1093/geroni/igaa057.2422

**Published:** 2020-12-16

**Authors:** Megumi Inoue, Emily Ihara, Catherine Tompkins, Kristen Suthers Rumrill, Kendall Barrett, Kathryn McNeil, Christi Clark

**Affiliations:** 1 George Mason University, Fairfax, Virginia, United States; 2 Harpeth Consultant Advisory Group, Franklin, Tennessee, United States

## Abstract

The Music & Memory intervention is a person-centered, non-pharmacologic intervention for people living with dementia. It is considered a personalized music intervention because the playlist comprises music genres that the individual prefers. Music has shown positive effects on behavioral and psychological symptoms of dementia. However, the findings from previous studies are often based on small sample sizes, insufficient descriptions of treatment fidelity, and lack of randomization. To address these issues, we began a randomized controlled trial implementing the Music & Memory intervention in nursing facilities in Virginia with support from the Centers for Medicare and Medicaid (CMS) Civil Money Penalty. We will present our research strategies as well as preliminary results suggesting that nursing facilities with higher occupancy rates are more likely to participate in this type of a program. In addition, study participants with dementia show positive emotional and behavioral reactions when listening to their favorite music.

